# Supplementary material for: The ESCRT protein CHMP5 promotes T cell leukemia by controlling BRD4-p300-dependent transcription
Source: bioRxiv. 2024 Jan 31:2024.01.29.577409. Preprint. [Version 1] doi: 10.1101/2024.01.29.577409 (PMC10862731; doi:10.1101/2024.01.29.577409)
Supplement: Supplement 1 [file NIHPP2024.01.29.577409v1-supplement-1.pdf]

bioRxiv preprint doi: https://doi.org/10.1101/2024.01.29.577409; this version posted January 31, 2024. The copyright holder for this preprint (which was not certified by peer review) is the author/funder. This article is a US Government work. It is not subject to copyright under 17 USC 105 and is also made available for use under a CC0 license.

Figure S1. CHMP5 promotes the T-ALL gene program, related to Figure 1

(A) CHMP5 mRNA and protein expression relative to Actin and normalized to CT CUTLL1 cells. Data are presented as average (± SD) of replicates pooled from 5 independent experiments. Student's t-test: ****, $p < 0.0001$.

(B) Viability of CT and KD CUTLL1 cells determined by Annexin-V and 7-AAD staining. Data points are technical replicates and representative of 2 experiments. Difference between CT and KD are not significant. ($p > 0.05$).

(C) EdU staining of CT and KD CUTLL1 cells with quantification of cells in different phases of cell cycle. Data are technical replicates and representative of 2 independent experiments. 2-way ANOVA: **, $p < 0.01$, ***, $p < 0.001$.

(D) Heatmaps of DEGs from MYC-target gene pathways.

(E) Western blot of CUTLL1 cells treated with cycloheximide (CHX) overtime with vehicle (DMSO) or 10 mM MG132. Lo, short exposure for MYC; Hi, long exposure for MYC.

(F) Quantification of MYC expression in DMSO and MG132 treated samples from (E) overtime, normalized to the 0-time point of each group. Data is representative of 2 experiments.

(G) Western blot of CT and KD CUTLL1 cells treated with vehicle (DMSO) or 10 mM MG132 for 6 hours.

(H) Mean fluorescence intensity (MFI) for ER Tracker and MitoSOX dyes in CT and KD CUTLL1 transduced with murine CHMP5 (mCHMP5) or vector control. Data points are technical

bioRxiv preprint doi: https://doi.org/10.1101/2024.01.29.577409; this version posted January 31, 2024. The copyright holder for this preprint (which was not certified by peer review) is the author/funder. This article is a US Government work. It is not subject to copyright under 17 USC 105 and is also made available for use under a CC0 license.

replicates. One-way ANOVA: **, $p < 0.01$; ns, $p > 0.05$

(I) mRNA expression of MYC-target metabolic genes. Data points are biological replicates from 3 independent experiments. One-way ANOVA: ****, $p < 0.0001$.

(J) Flow analysis of CUTTL1 cells treated with shRNAs for CT, CHMP5 or MYC. MFIs are normalized to CT. Data points are biological replicates from 2-3 independent experiments. One-way ANOVA: ****, $p < 0.0001$.

Figure S2. Identification of nuclear CHMP5-BRD4 interaction on chromatin, related to Figure 2

(A) Western blot of fractionated human T cells isolated from PBMCs. W, whole cell; C, cytoplasmic; and N, nuclear lysates. CHMP5 band intensity relative to the cytoplasmic band are indicated.

(B) Clustal Omega alignment of the N-terminal amino acid sequences of CHMP5 from different species. Sequences corresponding to the bipartite nuclear localization sequence (NLS) are highlighted.

(C) Amino acid sequence homology of CHMP5 from different species relative to human CHMP5.

(D) Western blot of fractionated CUTLL1 cells transduced with CHMP5-HA and subjected to immunoprecipitation with isotype (IgG) or anti-HA antibodies and immunoblotted for MYC and ICN1.

(E) Western blot of nuclear lysates in LOUCY cells subjected to immunoprecipitation with IgG or anti-BRD4 antibodies

(F) Nuclear lysate of HEK293T cells co-transfected with BRD4-FLAG or CHMP5-HA and subsequently immunoprecipitated with anti-FLAG antibodies and immunoblotted with anti-HA or anti-FLAG antibodies.

bioRxiv preprint doi: https://doi.org/10.1101/2024.01.29.577409; this version posted January 31, 2024. The copyright holder for this preprint (which was not certified by peer review) is the author/funder. This article is a US Government work. It is not subject to copyright under 17 USC 105 and is also made available for use under a CC0 license.

(G) Anti-FLAG immunoprecipitation of recombinant BRD4-FLAG and CHMP5-His recombinant protein.

(H) PCR of *RPL30* on antibody (Ab) immunoprecipitated chromatin DNA from CUTLL1 cells transduced with CHMP5-HA run on a 1% agarose gel.

Figure S3. CHMP5 mediates BRD4-driven Pol II pause release and super enhancer formation, related to Figure 3

(A) Pie chart of genome-wide BRD4 occupancy in control (CT) and CHMP5-depleted (KD) CUTLL1 cells.

(B) Metaplot of BRD4 binding at the TSS, gene body and TES across all genes in CT and KD CUTLL1 cells.

(C) Metaplot of Pol II binding at the TSS, gene body and TES across all genes in CT and KD cells.

(D) Box plot showing the ratio of KD to CT Pol II enrichment at the TES of UP and DOWN-regulated DEGs from RNA-seq on CT and KD CUTLL1 cells. Student's t-test: ****, $p < 0.0001$.

(E) Metaplot of H3K27ac density at the TSS, gene body and TES across active genes in CT and KD CUTLL1 cells.

(F) Pol II traveling ratio at active genes (defined by H3K27ac signal at promoter).

(G) Relative (normalized to control (CT) cells) mRNA expression of *MYC, TCF7, ERG,* and *ETV6* in CT and KD CUTLL1 cells. Data are average (± SD) of three biological replicates. Student's t-test: **, $p < 0.01$; ****, $p < 0.0001$.

(H) BRD4 and H3K27ac ChIP-seq tracks at the *ERG* and *ETV6* gene loci. SE, super-enhancer.

Figure S4. CHMP5 promotes the interaction between BRD4 and p300, related to Figure 4

bioRxiv preprint doi: https://doi.org/10.1101/2024.01.29.577409; this version posted January 31, 2024. The copyright holder for this preprint (which was not certified by peer review) is the author/funder. This article is a US Government work. It is not subject to copyright under 17 USC 105 and is also made available for use under a CC0 license.

(A) Relative mRNA (normalized to *ACTB*) and protein expression (normalized to Lamin B1) in CUTLL1 cells shown in **Figure 4A**. Data are average (± SD) of replicates from two independent experiments. Student's t-test: *, $p < 0.05$.

(B) Nuclear lysate from lentiviral CHMP5-HA-transduced CUTLL1 cells immunoprecipitated with isotype (IgG) or anti-HA antibody and immunoblotted for p300.

(C) Mechanistic model of CHMP5-mediated regulation of epigenetic and transcriptional program in T-ALL cells. In wildtype T-ALL cells (left), CHMP5 potentiates the p300-BRD4 interaction that mediates H3K27 hyperacetylation of *cis* enhancers and super-enhancers. Subsequent assembly of core transcriptional factors at promoters and enhancers enables proximal-promoter and distal enhancer interaction that stimulate Pol II pause-release and transcriptional elongation of pro-leukemogenic genes. By contrast, CHMP5 deficiency (right) impairs the p300-BRD4 interaction, which reduces H3K27 acetylation, and disrupts super-enhancer formation and interaction with proximal-promoters leading to impaired transcription of T-ALL genes.

Figure S5. CHMP5 expression and impact on human T-ALL prognosis and chemoresistance, related to Figure 5

(A) Relative CHMP5 protein expression in cancer cell lines in order from highest to lowest average. Data from Cancer Cell Line Encyclopedia (CCLE).

(B) Western blot of T-ALL cell lines and healthy donor human T-cells. Lysates from HEK293T (HEK) cells transduced with non-targeting shRNA (CT) or shCHMP5 (KD) lentivirus is used as control for anti-CHMP5 antibody specificity.

(C) Quantification of CHMP5 and VPS4 protein relative to Actin from (B). Student's t-test: ****, $p < 0.0001$.

(D-E) Overall survival of pediatric T-ALL patients (TARGET T-ALL) expressing high (top 20%)

bioRxiv preprint doi: https://doi.org/10.1101/2024.01.29.577409; this version posted January 31, 2024. The copyright holder for this preprint (which was not certified by peer review) is the author/funder. This article is a US Government work. It is not subject to copyright under 17 USC 105 and is also made available for use under a CC0 license.

and low (bottom 20%) levels of *VPS4A* (D) and *CHMP1A* (E).

(F) mRNA expression of *CHMP5* in pediatric T-ALL patients that achieved complete remission (CR) (n = 65) or did not achieve complete remission (NCR) (n = 4). Student's t-test: *, $p < 0.05$.

(G) Expression of *BIM* in CUTLL1 cells from **Figure 5J**. Data are mean (± SD) of technical replicates. One-way ANOVA: ****, $p < 0.0001$

(H) Viability of CT and KD CUTLL1 and LOUCY treated with JQ1 for 3 days. Data are presented as mean (± SD) of 3 technical replicates, representative of 2 independent experiments.

(I) IC_{50} for JQ1 in CUTLL1 and LOUCY cells. IC_{50} calculated by non-linear best-fit analysis. p-value calculated by 2-way ANOVA. FC, fold-change in CT versus KD IC_{50}.

Figure S6. CHMP5 deficiency impairs T-ALL development and progression in vivo, related to Figure 6

(A) Plasmid map of bi-cistronic ICN1 and NGFR expression retroviral plasmid. IRES, internal ribosomal entry site.

(B) Retrovirus-induced ICN1 leukemia mice experimental scheme.

(C) NGFR expression 48 hours after transduction of WT and KO donor BM cells.

(D) Hematoxylin and eosin staining of liver and spleen from leukemia mice. Scale bar = 200 µm

(E) Representative flow cytometry analysis of blood from leukemia mice at 4 weeks post-transplant with average (± SD) frequency of $CD45.2^{+}NGFR^{+}$ cells shown in graph. Student's t-test: ****, $p < 0.0001$; WT n=12, KO n=15 mice/group.

(F) Flow cytometry plots of CD4 and CD8 expression on $CD45.2^{+}NGFR^{+}$ cells with average frequency (± SD) of gated subsets in WT n = 4 and KO n = 5 mice. 2-way ANOVA: ****, $p < 0.0001$; WT, n = 4; KO, n = 5 mice.

bioRxiv preprint doi: https://doi.org/10.1101/2024.01.29.577409; this version posted January 31, 2024. The copyright holder for this preprint (which was not certified by peer review) is the author/funder. This article is a US Government work. It is not subject to copyright under 17 USC 105 and is also made available for use under a CC0 license.

(G) Average (± SD) mRNA expression of *Myc, C1qbp, Ldha,* and *Phb2* in splenic CD45.2$^+$NGFR$^+$ cells from WT (n = 8) and KO (n = 9) chimera mice. Expression is normalized to WT. Student's t-test, **p < 0.01, ****p < 0.0001.

(H) Venn-diagram of DEGs overlapping from CT and KD CUTLL1 (**Figure 1**), and WT and KO NGFR$^+$ splenocytes.

(I) Overlapping differentially expressed pathways from CT and KD CUTLL1, and WT and KO NGFR$^+$ splenocytes.

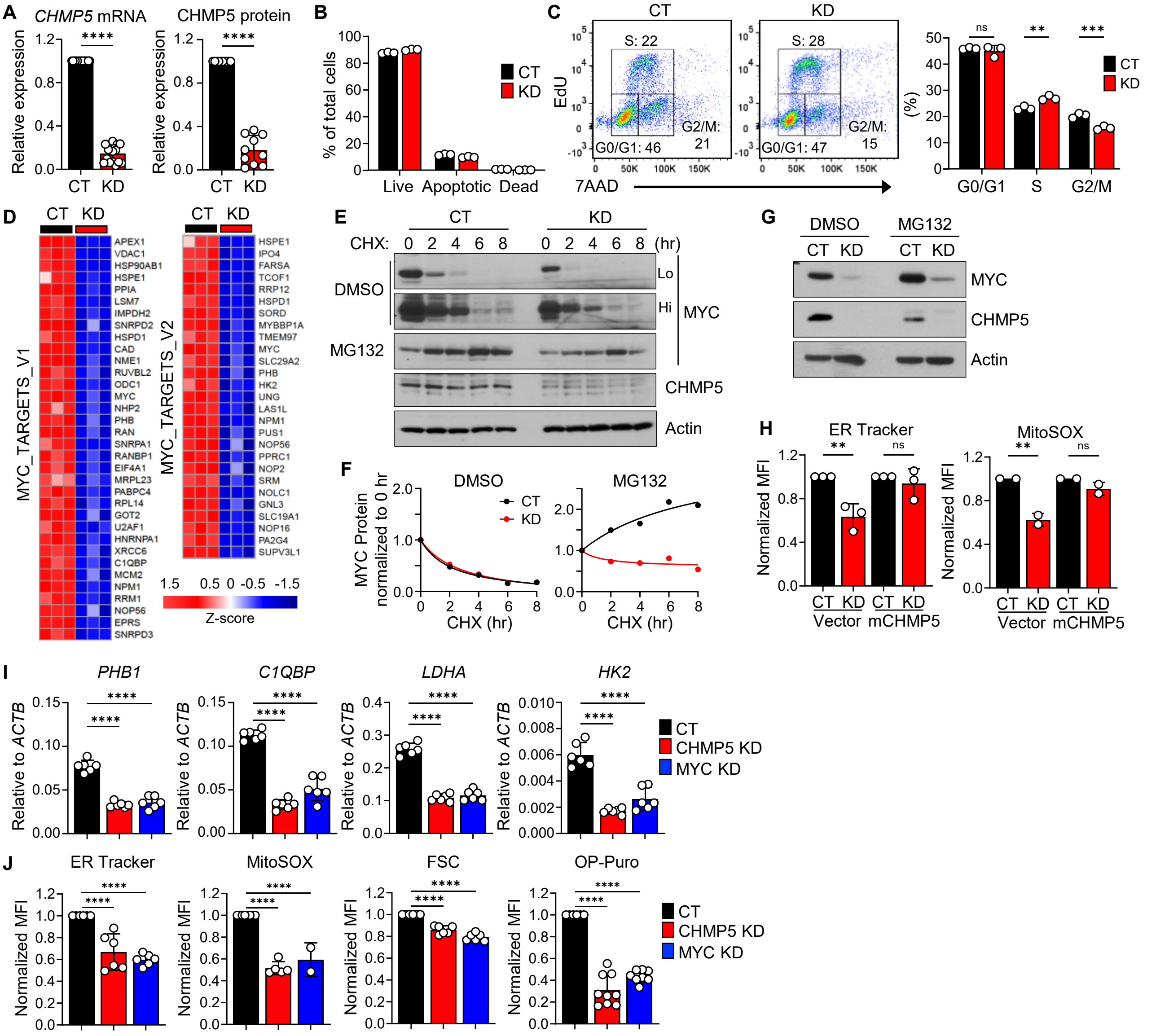

Figure S1. CHMP5 promotes the T-ALL gene program, related to Figure 1

(A) CHMP5 mRNA and protein expression relative to Actin and normalized to CT CUTLL1 cells. Data are presented as average (± SD) of replicates pooled from 5 independent experiments. Student's t-test: ****, $p < 0.0001$.

(B) Viability of CT and KD CUTLL1 cells determined by Annexin-V and 7-AAD staining. Data points are technical replicates and representative of 2 experiments. Difference between CT and KD are not significant. ($p > 0.05$).

(C) EdU staining of CT and KD CUTLL1 cells with quantification of cells in different phases of cell cycle. Data are technical replicates and representative of 2 independent experiments. 2-way ANOVA: **, $p < 0.01$, ***, $p < 0.001$.

(D) Heatmaps of DEGs from MYC-target gene pathways.

(E) Western blot of CUTLL1 cells treated with cycloheximide (CHX) overtime with vehicle (DMSO) or 10 µM MG132. Lo, short exposure for MYC; Hi, long exposure for MYC.

(F) Quantification of MYC expression in DMSO and MG132 treated samples from (**E**) overtime, normalized to the 0-time point of each group. Data is representative of 2 experiments.

(G) Western blot of CT and KD CUTLL1 cells treated with vehicle (DMSO) or 10 µM MG132 for 6 hours.

(H) Mean fluorescence intensity (MFI) for ER Tracker and MitoSOX dyes in CT and KD CUTLL1 transduced with murine CHMP5 (mCHMP5) or vector control. Data points are technical replicates. One-way ANOVA: **, $p < 0.01$; ns, $p > 0.05$

(I) mRNA expression of MYC-target metabolic genes. Data points are biological replicates from 3 independent experiments. One-way ANOVA: ****, $p < 0.0001$.

(J) Flow analysis of CUTTL1 cells treated with shRNAs for CT, CHMP5 or MYC. MFIs are normalized to CT. Data points are biological replicates from 2-3 independent experiments. One-way ANOVA: ****, $p < 0.0001$.

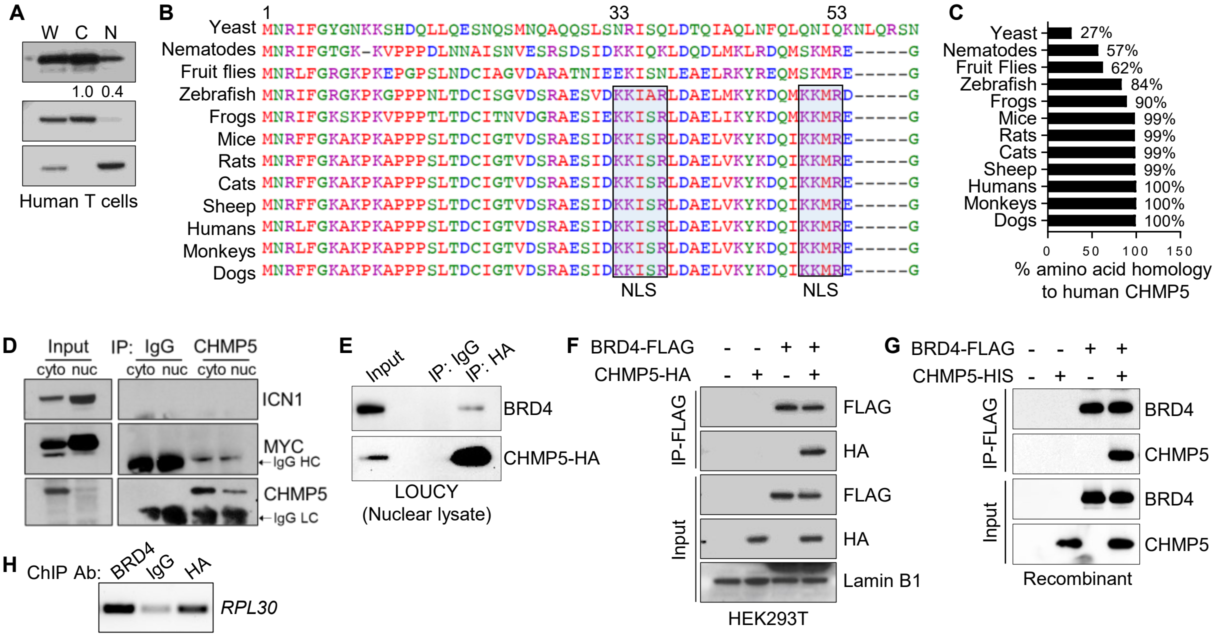

Figure S2. Identification of nuclear CHMP5-BRD4 interaction on chromatin, related to Figure 2

(A) Western blot of fractionated human T cells isolated from PBMCs. W, whole cell; C, cytoplasmic; and N, nuclear lysates. CHMP5 band intensity relative to the cytoplasmic band are indicated.

(B) Clustal Omega alignment of the N-terminal amino acid sequences of CHMP5 from different species. Sequences corresponding to the bipartite nuclear localization sequence (NLS) are highlighted.

(C) Amino acid sequence homology of CHMP5 from different species relative to human CHMP5.

(D) Western blot of fractionated CUTLL1 cells transduced with CHMP5-HA and subjected to immunoprecipitation with isotype (IgG) or anti-HA antibodies and immunoblotted for MYC and ICN1.

(E) Western blot of nuclear lysates in LOUCY cells subjected to immunoprecipitation with IgG or anti-BRD4 antibodies

(F) Nuclear lysate of HEK293T cells co-transfected with BRD4-FLAG or CHMP5-HA and subsequently immunoprecipitated with anti-FLAG antibodies and immunoblotted with anti-HA or anti-FLAG antibodies.

(G) Anti-FLAG immunoprecipitation of recombinant BRD4-FLAG and CHMP5-His recombinant protein.

(H) PCR of *RPL30* on antibody (Ab) immunoprecipitated chromatin DNA from CUTLL1 cells transduced with CHMP5-HA run on a 1% agarose gel.

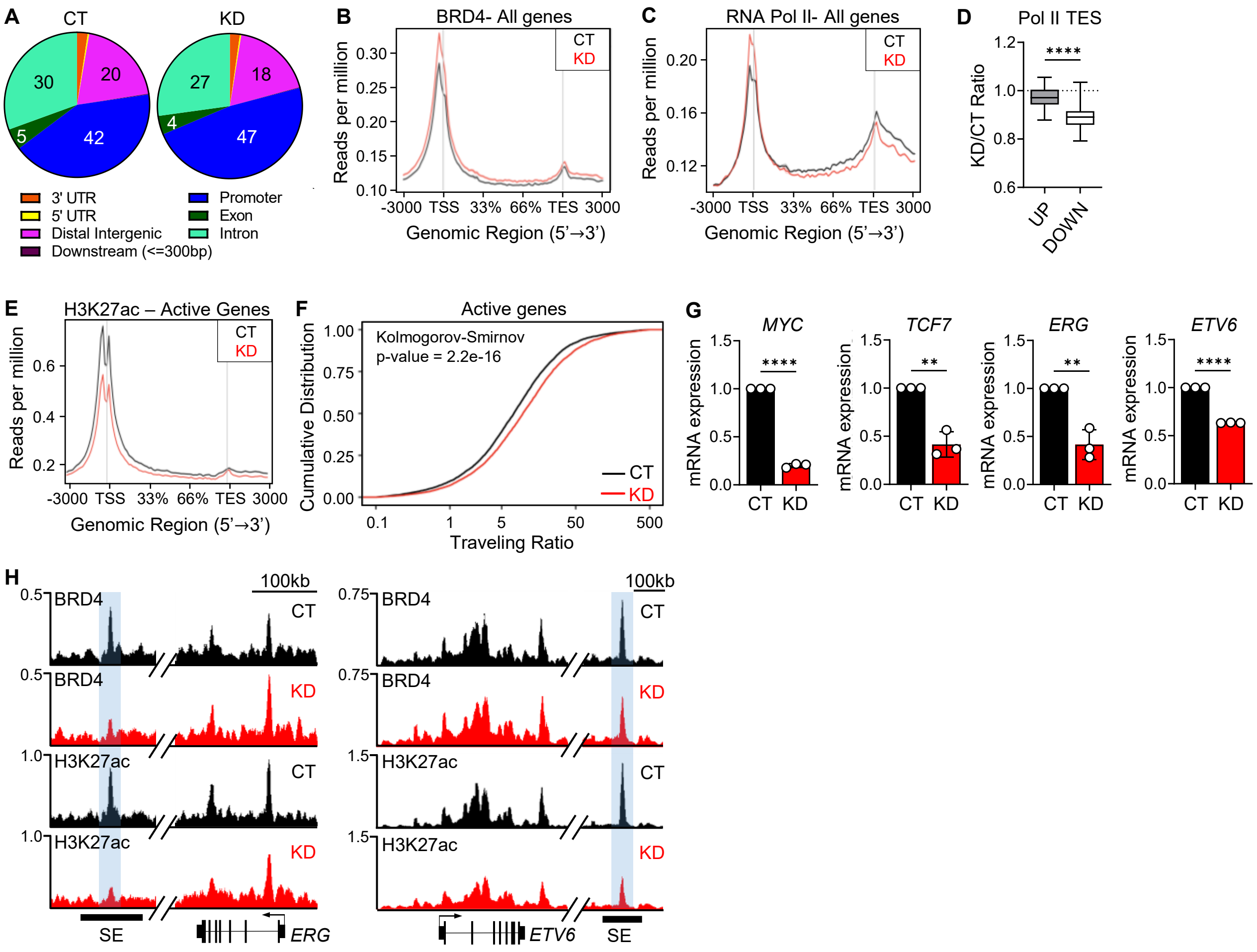

Figure S3. CHMP5 mediates BRD4-driven Pol II pause release and super enhancer formation, related to Figure 3

(A) Pie chart of genome-wide BRD4 occupancy in control (CT) and CHMP5-depleted (KD) CUTLL1 cells.

(B) Metaplot of BRD4 binding at the TSS, gene body and TES across all genes in CT and KD CUTLL1 cells.

(C) Metaplot of Pol II binding at the TSS, gene body and TES across all genes in CT and KD cells.

(D) Box plot showing the ratio of KD to CT Pol II enrichment at the TES of UP and DOWN-regulated DEGs from RNA-seq on CT and KD CUTLL1 cells. Student's t-test: ****, $p < 0.0001$.

(E) Metaplot of H3K27ac density at the TSS, gene body and TES across active genes in CT and KD CUTLL1 cells.

(F) Pol II traveling ratio at active genes (defined by H3K27ac signal at promoter).

(G) Relative (normalized to control (CT) cells) mRNA expression of *MYC, TCF7, ERG,* and *ETV6* in CT and KD CUTLL1 cells. Data are average (± SD) of three biological replicates. Student's t-test: **, $p < 0.01$; ****, $p < 0.0001$.

(H) BRD4 and H3K27ac ChIP-seq tracks at the *ERG* and *ETV6* gene loci. SE, super-enhancer.

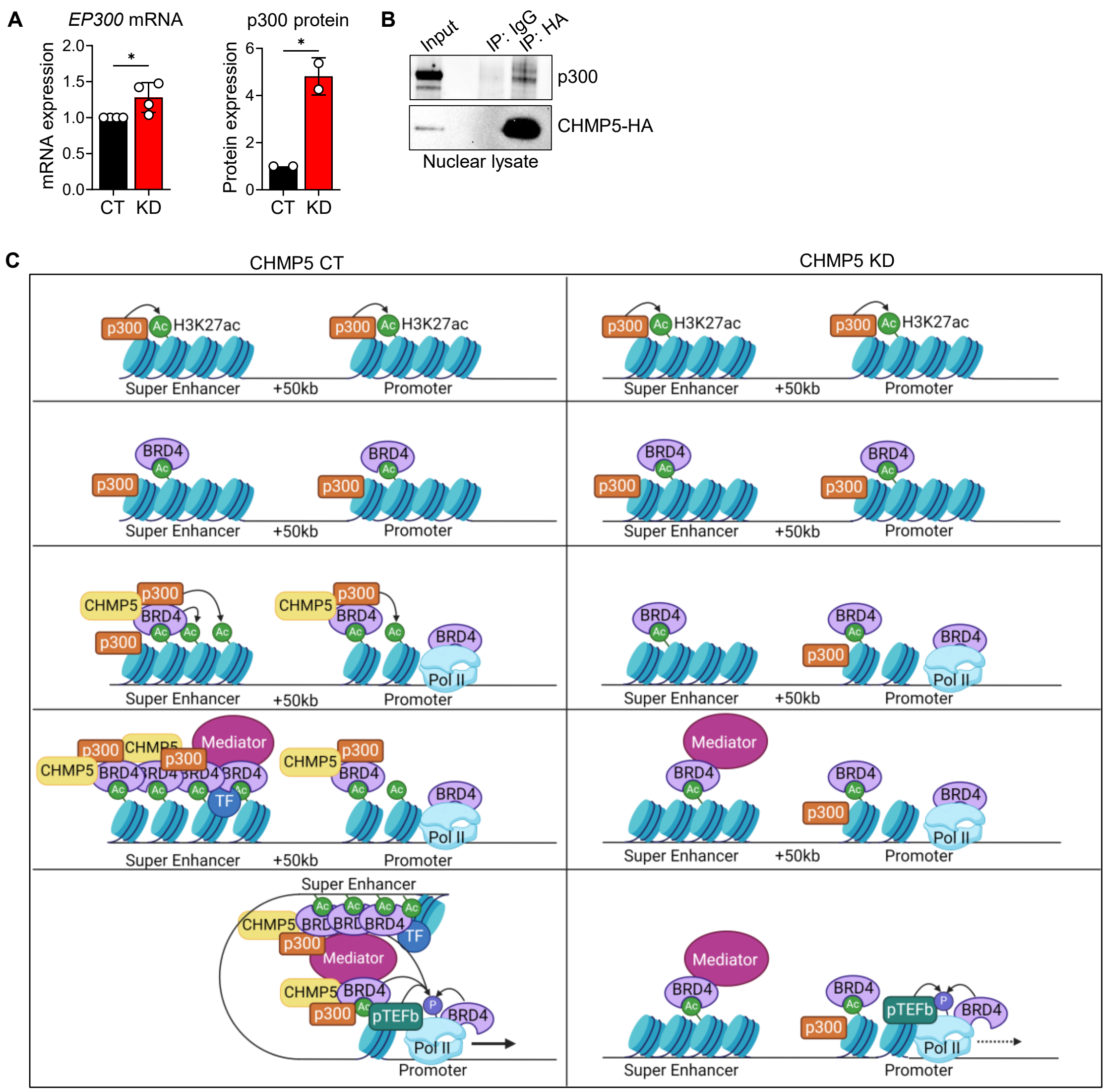

Figure S4. CHMP5 promotes the interaction between BRD4 and p300, related to Figure 4

(A) Relative mRNA (normalized to *ACTB*) and protein expression (normalized to Lamin B1) in CUTLL1 cells shown in **Figure 4A**. Data are average (± SD) of replicates from two independent experiments. Student's t-test: *, $p < 0.05$.

(B) Nuclear lysate from lentiviral CHMP5-HA-transduced CUTLL1 cells immunoprecipitated with isotype (IgG) or anti-HA antibody and immunoblotted for p300.

(C) Mechanistic model of CHMP5-mediated regulation of epigenetic and transcriptional program in T-ALL cells. In wildtype T-ALL cells (left), CHMP5 potentiates the p300-BRD4 interaction that mediates H3K27 hyperacetylation of *cis* enhancers and super-enhancers. Subsequent assembly of core transcriptional factors at promoters and enhancers enables proximal-promoter and distal enhancer interaction that stimulate Pol II pause-release and transcriptional elongation of pro-leukemogenic genes. By contrast, CHMP5 deficiency (right) impairs the p300-BRD4 interaction, which reduces H3K27 acetylation, and disrupts super-enhancer formation and interaction with proximal-promoters leading to impaired transcription of T-ALL genes.

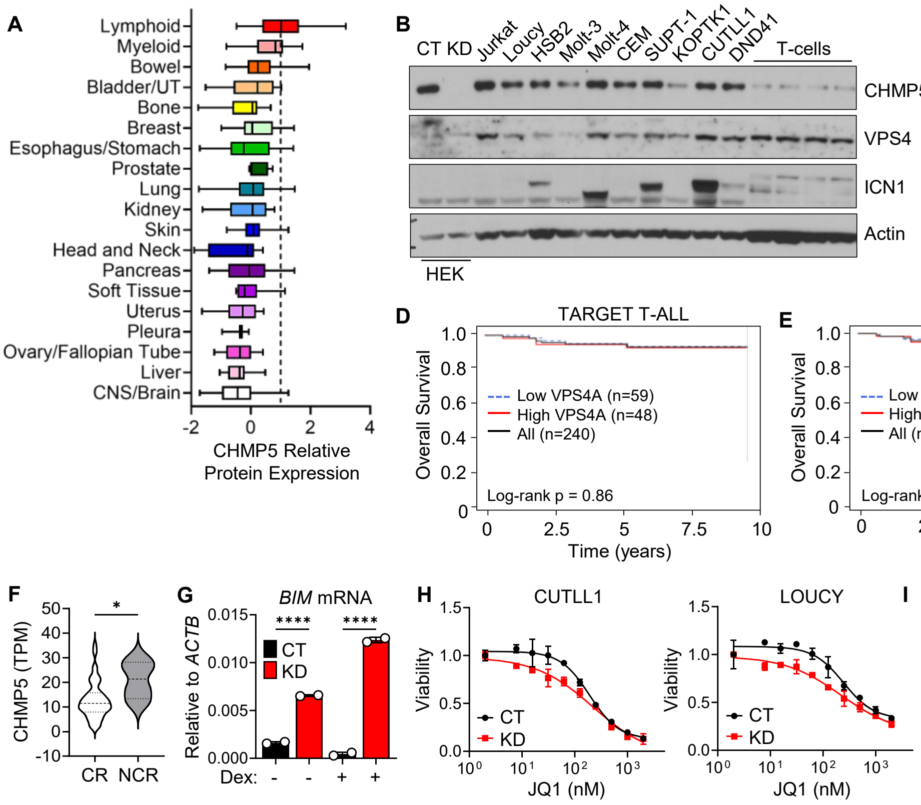

JQ1	CUTLL1	LOUCY
CT IC_{50}	241 nM	245 nM
KD IC_{50}	197 nM	223 nM
FC	1.2x	1.1x
p-value	ns	ns

Figure S5. CHMP5 expression and impact on human T-ALL prognosis and chemoresistance, related to Figure 5

(A) Relative CHMP5 protein expression in cancer cell lines in order from highest to lowest average. Data from Cancer Cell Line Encyclopedia (CCLE).

(B) Western blot of T-ALL cell lines and healthy donor human T-cells. Lysates from HEK293T (HEK) cells transduced with non-targeting shRNA (CT) or shCHMP5 (KD) lentivirus is used as control for anti-CHMP5 antibody specificity.

(C) Quantification of CHMP5 and VPS4 protein relative to Actin from (B). Student's t-test: ****, $p < 0.0001$.

(D-E) Overall survival of pediatric T-ALL patients (TARGET T-ALL) expressing high (top 20%) and low (bottom 20%) levels of *VPS4A* (D) and *CHMP1A* (E).

(F) mRNA expression of *CHMP5* in pediatric T-ALL patients that achieved complete remission (CR) (n = 65) or did not achieve complete remission (NCR) (n = 4). Student's t-test: *, $p < 0.05$.

(G) Expression of *BIM* in CUTLL1 cells from **Figure 6J**. Data are mean (± SD) of technical replicates. One-way ANOVA: ****, $p < 0.0001$

(H) Viability of CT and KD CUTLL1 and LOUCY treated with JQ1 for 3 days. Data are presented as mean (± SD) of 3 technical replicates, representative of 2 independent experiments.

(I) IC_{50} for JQ1 in CUTLL1 and LOUCY cells. IC_{50} calculated by non-linear best-fit analysis. p-value calculated by 2-way ANOVA. FC, fold-change in CT versus KD IC_{50}.

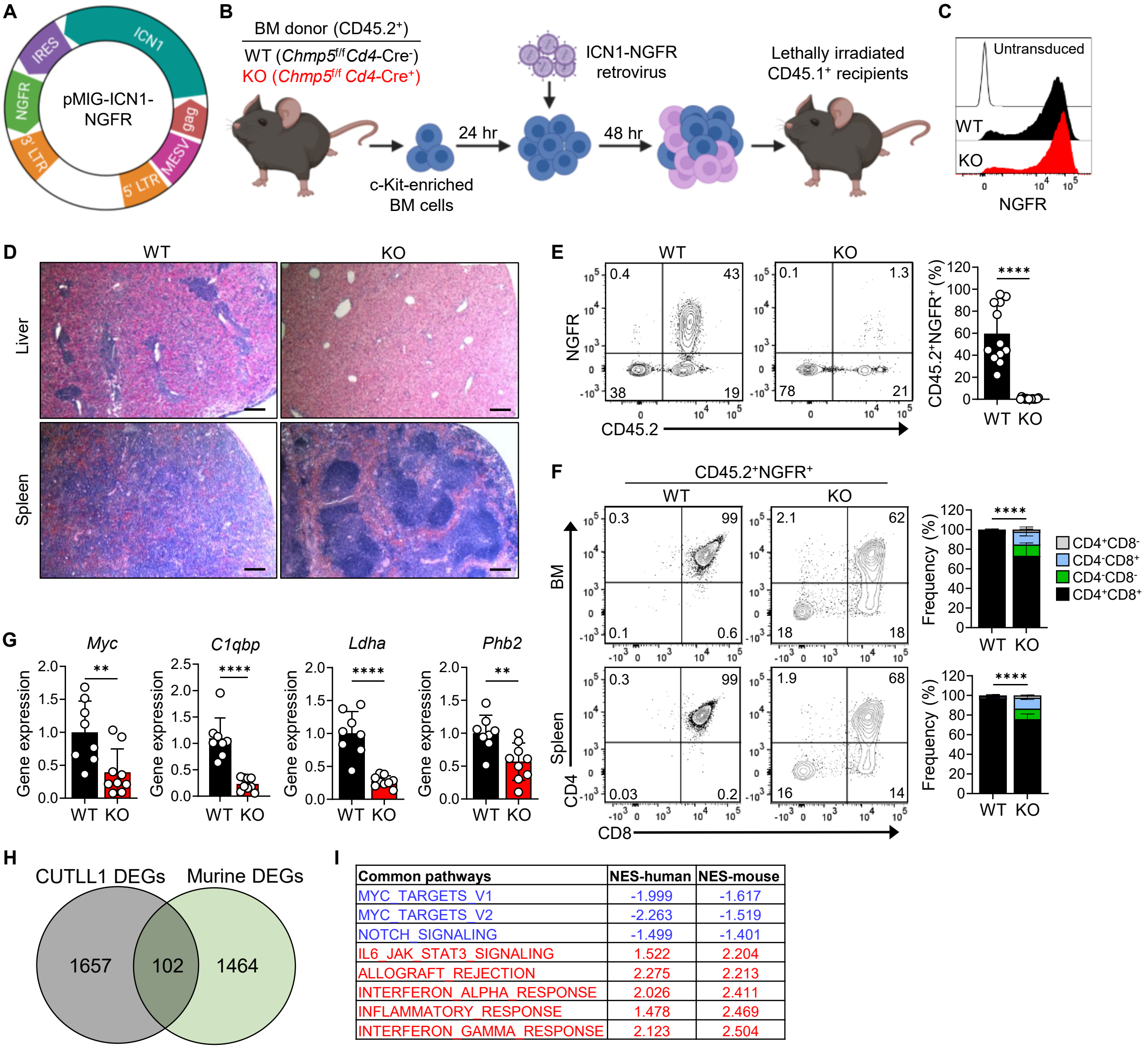

Common pathways	NES-human	NES-mouse
MYC_TARGETS_V1	-1.999	-1.617
MYC_TARGETS_V2	-2.263	-1.519
NOTCH_SIGNALING	-1.499	-1.401
IL6_JAK_STAT3_SIGNALING	1.522	2.204
ALLOGRAFT_REJECTION	2.275	2.213
INTERFERON_ALPHA_RESPONSE	2.026	2.411
INFLAMMATORY_RESPONSE	1.478	2.469
INTERFERON_GAMMA_RESPONSE	2.123	2.504

Figure S6. CHMP5 deficiency impairs T-ALL development and progression in vivo, related to Figure 6

(A) Plasmid map of bicistronic ICN1 and NGFR expression retroviral plasmid. IRES, internal ribosomal entry site.

(B) Retrovirus-induced ICN1 leukemia mice experimental scheme.

(C) NGFR expression 48 hours after transduction of WT and KO donor BM cells.

(D) Hematoxylin and eosin staining of liver and spleen from leukemia mice. Scale bar = 200 µm

(E) Representative flow cytometry analysis of blood from leukemia mice at 4 weeks post-transplant with average (± SD) frequency of CD45.2$^+$NGFR$^+$ cells shown in graph. Student's t-test: ****, $p < 0.0001$; WT n=12, KO n=15 mice/group.

(F) Flow cytometry plots of CD4 and CD8 expression on CD45.2$^+$NGFR$^+$ cells with average frequency (± SD) of gated subsets in WT n = 4 and KO n = 5 mice. 2-way ANOVA: ****, $p < 0.0001$; WT, n = 4; KO, n = 5 mice.

(G) Average (± SD) mRNA expression of *Myc, C1qbp, Ldha,* and *Phb2* in splenic CD45.2$^+$NGFR$^+$ cells from WT (n = 8) and KO (n = 9) chimera mice. Expression is normalized to WT. Student's t-test, **$p < 0.01$, ****$p < 0.0001$.

(H) Venn-diagram of DEGs overlapping from CT and KD CUTLL1 (**Figure 1**), and WT and KO NGFR$^+$ splenocytes.

(I) Overlapping differentially expressed pathways from CT and KD CUTLL1, and WT and KO NGFR$^+$ splenocytes.
